# Marginal permeability of one step self-etch adhesives: Effects of double application or the application of hydrophobic layer

**DOI:** 10.4103/0972-0707.71646

**Published:** 2010

**Authors:** R Pushpa, B S Suresh

**Affiliations:** Department of Conservative and Endodontics, Kothiwal Dental College and Research Centre, Moradabad, Uttar Pradesh, India; 1 Department of Pedodontics, Teerthanker Mahaveer Dental College and Research Centre, Moradabad, Uttar Pradesh, India

**Keywords:** Double application, hydrophobic layer, marginal permeability, self-etch adhesives

## Abstract

**Aim:**

The purpose of this in vitro investigation was to evaluate the influence of double application and application of hydrophobic layer on marginal adaptation of four self-etch adhesive systems (XENOIII, ALLBONDSE, CLEARFIL SE TRI BOND, FUTURA BOND).

**Materials and Methods:**

One hundred and twenty class V cavities were prepared on intact, extracted human premolars and were divided into three groups of ten teeth each for all four adhesives. Group 1: Application of bonding agents as per manufacturer directions. Group 2: Double application of bonding agents. Group 3: Application of hydrophobic layer. The specimens were restored with composite and light cured. After thermocycling and immersion in 2% Basic Fuchsin dye solution, the teeth were sectioned and dye penetration was observed under a stereomicroscope at 20× magnification. All the samples were scored and results were analyzed using Kruskal-Wallis and Mann-Whitney tests.

**Results:**

Group 3, in which the adhesive systems were coated with hydrophobic layer, showed significantly decreased microleakage, followed by Group 1 and Group 2 for all the adhesive systems. And there is no significant different between Group 1 and Group 2.

**Conclusion:**

Marginal permeability of one-step adhesives can be minimized by the application of more hydrophobic resin layer, and the double application of one-step self-etch system can be safely performed without jeopardizing the performance of adhesives.

## INTRODUCTION

Adhesion to dentin has been the subject of considerable interest over the last few decades. And dentin bonding agents have undergone dramatic changes in chemistry and clinical use over the last few decades. Adhesion is required to oppose and withstand the contraction forces during polymerization of composite resin and insure retention and marginal integrity during functioning of the restored teeth[1] Depending on the configuration of the restoration, material properties and application technique, the bonded restoration is challenged by stress which may exceed the adhesive or cohesive strength.[2]

To reduce technique sensitivity that affects the bonding ability of adhesive systems, steps required for bonding procedures have been reduced.[3] New approaches for bonding restorative material to tooth substrates without phosphoric-acid etching, such as self-etch systems, have been introduced. Recently, single-step self-etch adhesive systems that combine the function of a self-etching primer and a bonding agent have been developed.[4] The use of single step self-etch adhesives may eliminate technique-sensitive factors that negate the bonding ability of the restorations. The single-step self-etch adhesive is applied to the tooth surface prior to resin composite placement to ensure maximum adhesion through the mechanism of improved monomer penetration into the tooth substrate as well as improved wet ability of the tooth surface via the resin components. The use of single-step adhesives could also eliminate the possibility of discrepancies between the depth of etching and resin monomer penetration.[5]

As these materials have become popular among clinicians, they are being introduced at a very fast rate, without comprehensive testing to substantiate their performance.

Although very simple in technique, studies show that these systems may not perform as well as two- step self- etching priming system and etch-and-rinse systems. This inferior performance has been attributed to certain factors. First, the etching pattern of self etch adhesives is not as well defined as that provided by phosphoric acid.[6–8] Second, these products create very thin coatings,[9–11] which may be oxygen inhibited, resulting in a poorly polymerized adhesive layer.[12] Third, they are highly prone to phase separation as the solvent evaporates from the solution and finally they behave as permeable membranes after polymerization.[13] Many authors remarked that among all the classes of dentin adhesives available, none of the contemporary one-step self-etch systems could compete with more traditional multi-step adhesives.

Thus, the objective of this study is to evaluate the marginal adaptation of self-etch adhesives with two alternative modes of application (double coat and placement of a hydrophobic resin coat) and compare them with the manufacturer’s directions.

This study tested the null hypothesis that there is no effect of double- application or the application of hydrophobic layer on marginal permeability of one-step self-etch adhesives.

## MATERIALS AND METHODS

Four one step self- etch adhesive systems XENO-III (XE, denstply de trey, konstanz, Germany), ALL-BOND SE (Bisco, inc, Schaumburg, U.S.A), CLEARFILTRI-SBOND (Kuraray medicals), FUTURABOND NR (Voco, Cuxhaven, Germany) and Filtek^™^ Z250 (3M ESPE) hybrid composite material were used.

Sixty intact caries- free human premolar extracted for orthodontic purpose were selected. Any extrinsic stains or calculus deposits on teeth were cleaned using ultrasonic scaler and specimens were stored in isotonic saline until used. Class V cavity with a dimension of 3mm incisogingivally, 3mm mesiodistally and 1.5 mm depth was prepared on both buccal and lingual surfaces of all teeth for total of 120 cavities. The preparations were made with no 245 carbide bur in high- speed, under copious water coolant. After every 5 preparation, the bur was discarded and replaced with new one. The enamel margins were given 0.5mm bevel at 45° angle by using tapered fissure bur. The specimens were randomly and equally assigned to 3 experimental groups (n=10) for each of “all in one” adhesives, as shown in Table 1. Adhesive systems, composition and application modes of different groups are described in Table 2.

The restored specimens were stored in distilled water for 10 days. Then specimens were thermocycled for 1,000 cycles at 5° ± 1° and 55° ±1° with 30 sec of dwell time.

**Table 1:** Distribution of samples

Group 1	Application of bonding agents as by manufacturer directions
Group 2	Double-Application of bonding agents
Group 3	Application of hydrophobic layer

**Table 2:** Adhesive systems - composition and application mode of different groups

Adhesive system manufaturer	Composition	Group 1 manufacture direction (Md)	Group 2 double application(da)	Group 3 hydrophobic resin layer (Hr)
XENO 111 (Dentsply) Two bottles	Liquid A:HEMA, ethanol, water, aerosol, stabilizer; Liquid B: Pyro-EMA, pem-fudma conforquinone stabilizers, Eethyl- 4 Dimethyl aminobenzoate (coinitiator)	Mix liquids A&B for 5 s; Application of one thick coat of adhesive under pressure(30s); Gentle air stream(10s at 20cm); Light-activation (10s-600mw/cm^2^) Composite placement curing-40s	Steps 1-3 from (MD) Repeat steps 2-3; Step 4. Step 5.	Step 1-4 from (MD) Application of one coat of liquid B Air stream to make the bond film uniform(3s at 20cm) Light activation(10s-600 mw/cm^2^) Composite curing 40 s
All bond se (Bisco) Two bottles	Part1-ethanol, water, sodium benzene sulfinate; Part11-Hydroxyethyl methacrylate, Bis (glyceryl, 1,3 dimethyacrylate) phosphate, Biphenyldimethacrylate.	Mix liquids I&II until uniform pink; Application of one thick coat of adhesive under pressure(10s); Gentle air stream(10s at 20cm); Light-activation (10s-600mw/cm^2^) Composite placement curing-40s	Steps 1-3 from (MD) Repeat steps 2-3; Step 4. Step 5.	Step 1-4 from (MD) Application of one coat of liquid B (of XENOIII) Air stream to make the bond film uniform(3s at 20cm) Light activation(10s 600 mw/cm^2^) Composite curing 40 s
Clearfil sebond (Kuraray) Single bottle	MDP, bis-GMA, HEMA, initiator, ethanol, water, stabilizer, filler, hydrophobic dimethacrylate	Application of one coat of adhesive(20s) Gental air stream (10s) Light-activation (10s-600mw/cm^2^) Composite placement curing-40s	Steps 1-2 from (MD) Repeat steps 1-2 Step 3. Step 4.	Step 1-3 from (MD) Application of one coat of liquid B (of XENOIII) Air stream to make the bond film uniform(3s at 20cm) Light activation(10s -600 mw/cm^2^) Composite curing 40 s
Futura bond nt (Voco) Two bottles	Organic acids Bis-Gma, HEMA, TEMPTMA, compherchinon, amines (DABE), BHT, fluorides, ethanol.	Mix liquids A&B for 5s; Application of one thick coat of adhesive under pressure(30s); Gentle air stream(5s at 0cm); Light-activation (10s-600mw/cm^2^) Composite placement curing-40s	Steps 1-3 from (MD) Repeat steps 2-3; Step 4. Step 5.	Step 1-4 from (MD) Application of one coat of liquid B (of XENOIII) Air stream to make the bond film uniform(3s at 20cm) Light activation(10s 600 mw/cm^2^) Composite curing 40 s

The teeth surfaces were painted with two layers of nail varnish, except for a 1mm rim around the margin. To prevent any dye leakage through apical foramen, it was sealed with yellow sticky wax. All the specimens were immersed in freshly prepared 2% solution of “basic fuchsin dye” for 24 h in separate containers and were labeled for identification. After drying the samples at room temperature, the teeth were sectioned buccolingually with the help of diamond disc held in a straight hand-piece; all the samples were observed under stereomicroscope (Nikon SMZ1000) at 20X magnifications and images of sections were taken. The following ranking systems were used to score degree of dye penetration.[14]

0-No dye penetration; 1-Dye penetration up to half of the cavity depth; 2- Dye penetration more than the half of the cavity depth, but not extending the axial wall; 3- Dye penetration arriving to the cavity floor/axial wall and beyond. The data were analyzed using non- parametric Kruskal-Wallis (*P*<0.05) and Mann-Whitney test (*P*<0.05) in SPSS 15 software package.

## RESULTS

Table 3 shows the mean scores of microleakage of Xeno III, All Bond SE, Clearfil tri-s bond, Futurabond NR. Group 3, in which the adhesive systems were coated with hydrophobic layer, showed significantly decreased microleakage, then the Group 1 and Group 2 for all the adhesive systems. And there is no significant difference between group 1 and group 2 and group 3 for all other adhesives except for Xeno 111. For Xeno 111, Group 3 showed significantly decreased microleakage than Group 2.

**Table 3:** Mean scores for dye penetration of all the four adhesives

Adhesive	Group 1	Group 2	Group 3
Xeno III	2.2	1.6	0.5
Allbond Se	1.9	1.4	0.6
Clearfil S3bond	2.1	1.5	0.7
Futurabond Nr	2.0	1.6	0.6

## DISCUSSION

Difficulty encountered with adhesive restorative procedures is the impaired marginal seal, which allows access of bacteria, oral fluids, molecules and ions at the preparation walls/restorative material interface.[15]

To ensure outstanding marginal adaptation of an adhesive restoration, some restorative aspects must be considered, such as preparation design,[16] resin composite shrinkage,[17] and dental adhesive bonding strengths.[15]

Numerous studies are evident as regards to shear bond strength and microleakage, which being the two characteristics of adhesives that are frequently evaluated in vitro studies of dentin bonding systems.

In this study, dye microleakage method was employed to test the performance of self-etch adhesives under different mode of application.

Results of this study showed that, marginal adaptation of the all the self etch adhesive systems was affected by mode of application. Better marginal adaptation of the samples in which double application was observed. This was also observed by other authors Ries A, Albuquerque M *et al*,[18] Hashimoto M, Sano H, Yoshida E *et al*.[11,19]

Several mechanisms could account for the better performance of double application. As the first layers of the adhesive begins to etch the dentin substrate, it might become rapidly buffered by the hydroxyapatite,[20] so that the additional layers of unpolymerized acidic monomers may improve the etching ability of these adhesives by increasing the concentration of acidic reagents. Simultaneously to this process, more impregnation of resin might occur by additional supply of adhesive resin, as hypothesized by Ito *et al*.[11]

In this study, liquid B of XENOIII was used as hydrophobic layer in group 3; this was in accordance with Brackett, Tay and others.[21]

When comparing the adhesive systems that were applied according to manufacturer’s recommendation, the marginal adaptation of all the adhesive systems was significantly improved in samples in which additional hydrophobic layer was applied. Several mechanisms could account for the better performance of this group. This finding is in accordance with the study conducted by Pashley and others; they found higher *µ*TBS for Adper Prompt when a second adhesive layer was applied, followed by polymerization of the first layer. The author observed that an additional application of the bonding agent could seal the non- polymerized layer oxygen, thus enabling it to be adequately polymerized.[22] The additional hydrophobic layer must have increased the concentration of the hydrophobic monomer, reducing the relative concentration of solvents and hydrophilic monomers within the adhesive interface,[23–25] thus reducing the intrinsic permeability of these one step self-etching adhesives.

This hydrophobic layer seems to limit diffusion of water through the hybrid layer to the interface between the adhesive and resin composite (prevents the formation of water blisters), which could otherwise have occurred rapidly.[26–28] That could inhibit polymerization and thereby weaken the adhesive/resin composite interface.[29]

Zones of poorly polymerized hydrophilic phases that permit water movement have been demonstrated within the hybrid layers and self-etching adhesives. This hydrophobic layer may also have slowed the extraction of unpolymerized monomers or oligomers from the hybrid layer.[30]

This additional layer of resin increased the thickness of the adhesive layer, which is known to reduce polymerization stress and improve the marginal seal.[31]

Both the double application and the use of hydrophobic layer coating allowed the achievement of higher or at least equal performance of adhesives than the manufacturer’s direction. In other words, this means that no detrimental effect is to be observed when these techniques are used regardless the adhesive systems. Therefore, the null-hypothesis has to be rejected.

## CONCLUSION

Within the limitation of this study, it can be concluded that the marginal permeability of one-step adhesives can be minimized by the application of more hydrophobic resin.
